# Management of Delayed Complications of Hyaluronic Acid Fillers: Case Series From the Middle East

**DOI:** 10.1111/jocd.70166

**Published:** 2025-04-21

**Authors:** Yara Saad, Zeina Tannous

**Affiliations:** ^1^ Department of Dermatology Lebanese American University Gilbert and Rose‐Marie Chagoury School of Medicine Beirut Lebanon; ^2^ Department of Dermatology Wellman Center for Photomedicine, Massachusetts General Hospital, Harvard Medical School Boston Massachusetts USA

**Keywords:** complications, dermal filler, granuloma, hypersensitivity, infection, patient management

## Abstract

**Background:**

Hyaluronic acid (HA) dermal fillers are primarily utilized for restoring volume loss and for the correction of wrinkles and folds. While HA injections are generally considered safe, undesirable and serious complications have been on the rise due to the increased frequency of these procedures. Late‐onset complications involve filler migration, foreign body granuloma reactions, delayed hypersensitivity reactions, and infections, among others.

**Aims/Objectives:**

Raising awareness about late complications associated with HA filler injections and highlighting proper management strategies.

**Methods:**

We hereby present three patients who experienced distinct forms of delayed reactions to HA fillers, provide a systematic approach to their evaluation, and outline effective treatment strategies to ensure optimal patient outcomes. Written informed consent was obtained from the patients for publication of the details of their medical cases and any accompanying images.

**Results:**

Delayed complications to HA fillers include erythema, edema, inflammation, nodules, and infections. These late‐onset reactions present weeks to months post‐injection and may be influenced by immune responses, filler properties, and procedural factors. Ultrasound imaging is a valuable tool in diagnosing and managing these complications, guiding treatments such as hyaluronidase and corticosteroid injections. Preventive measures, including aseptic techniques, patient screening, and proper injector training, help in reducing risks and optimizing patient outcomes.

**Conclusion:**

HA filler injections remain among the most popular cosmetic procedures, generally offering safe outcomes when performed by skilled professionals. Complications may still occur and require prompt recognition and management. This article highlights the importance of imaging in diagnosing non‐ischemic complications and provides practical recommendations for managing delayed HA filler reactions. Ensuring patient safety relies on proper training, adherence to aseptic techniques, and careful patient and product selection.

## 1 Introduction

Hyaluronic acid (HA) dermal fillers, also known as soft tissue fillers, are primarily utilized for the correction of wrinkles and folds, as well as for restoring soft tissue volume loss caused by aging and certain medical conditions [1]. The use of HA dermal fillers has surged in recent years, with 5 416 274 injections reported in 2019 by the American Society of Plastic Surgeons and the American Society for Dermatologic Surgery—representing a 53% increase since 2012 [2]. These fillers have been rated the second most popular non‐surgical cosmetic procedure, following botulinum toxin injections [3].

An ideal injectable material should be able to provide a good aesthetic outcome with long‐lasting effects, ensuring safety, biocompatibility, and stability at the injection site with minimal risk of complications and migration [4]. While HA dermal fillers are generally considered safe, undesirable and serious complications have been on the rise in tandem with the increase in the number of injections [5]. Side effects are typically categorized into early onset (days to weeks from the time of injection) and late onset (weeks to years). Early onset complications may include bruising, edema, hypersensitivity reactions, the Tyndall effect, asymmetry and irregularities, infections, and vascular compromise [6, 7]. Late‐onset complications can involve filler migration, foreign body granuloma reactions, delayed hypersensitivity reactions, and infections, among others [6, 7, 8].

We hereby present three patients who experienced distinct forms of delayed reactions to HA fillers, with the aim of raising awareness of some of the late complications associated with soft‐tissue filler injections.

## 2 Case Reports

### 2.1 Case 1

**FIGURE 1:**
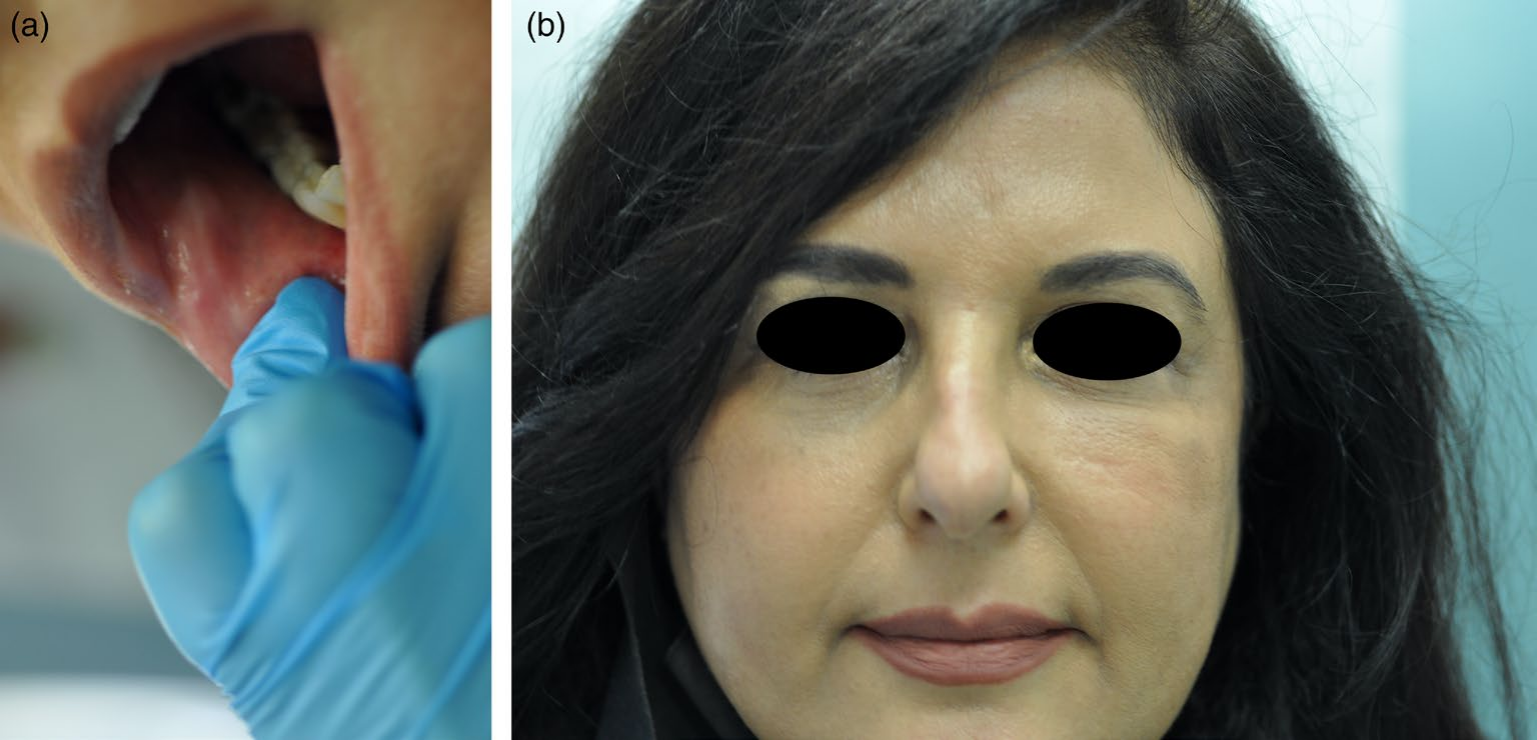
Clinical photographs of case 1. (a) Intra‐oral view of the subcutaneous nodules on the inferior cheeks. (b) Nodules emerging on the left infra‐orbital cheek.

A 56‐year‐old Lebanese female patient presented with firm subcutaneous nodules on the corners of the mouth and inferior nasolabial cheeks for 3 days, associated with a sensation of tightness or reduced range of motion over these areas. The lumps were not easily visible to the naked eye but could be readily felt through the buccal mucosa (Figure 1a). She had received HA soft tissue filler injections to the nasolabial folds and labio‐mandibular folds using Teosyal RHA4 (Teoxane S.A., Geneva, Switzerland) as well as the tear‐trough areas using Teosyal PureSense Redensity [II] (Teoxane S.A., Geneva, Switzerland) 3 months earlier by the author. She did not experience any other signs or symptoms such as fever or pain.

**FIGURE 2:**
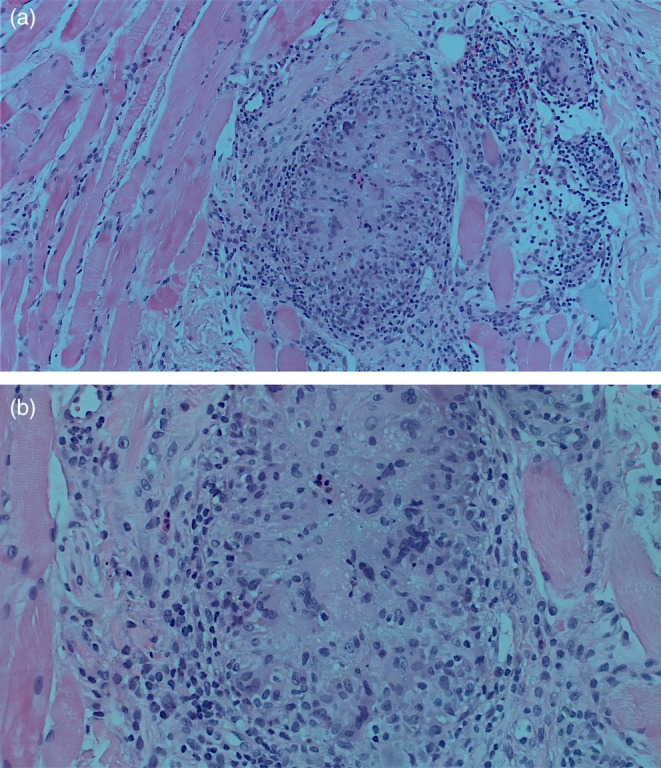
Biopsy results of patient 1 demonstrating a foreign body granulomatous reaction with giant cell formation, dissecting into the skeletal muscles. (a) H&E, low power (b) H&E, high power.

**FIGURE 3:**
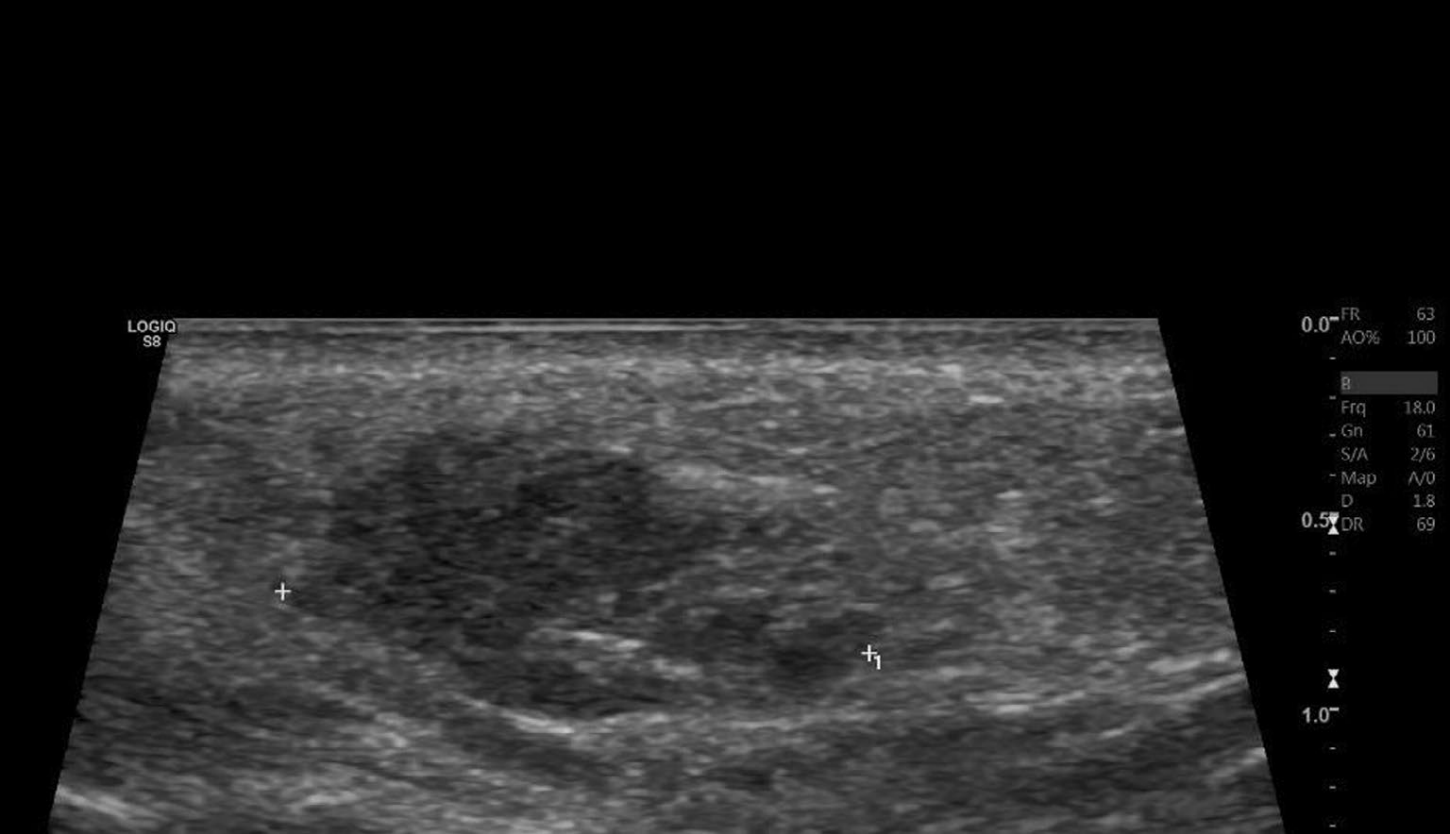
Ultrasonographic findings in case 1 demonstrating bilateral lobulated hypoechoic nodules within the subcutaneous tissues of the nasolabial folds, most likely representing a granulomatous reaction to soft tissue fillers.

She was initially treated with a course of oral prednisone, starting at 60 mg daily (equivalent to 1 mg/kg) and tapered over the course of 1 week. This resulted in only minimal improvement, along with the emergence of new nodules on her infra‐orbital cheeks (Figure 1b), prompting the decision to perform a punch biopsy. While waiting for the biopsy results, hyaluronidase injections were also administered at a concentration of 150 IU/mL, leading to more reduction in the size of the nodules. The biopsy results subsequently showed a foreign body granulomatous reaction, dissecting into the skeletal muscles (Figure 2). An ultrasound was also performed and revealed subcutaneous nodules consistent with granuloma formation (Figure 3). As a result, intralesional triamcinolone was injected at a dose of 10 mg/mL, which ultimately resulted in the complete resolution of the lumps.

### 2.2 Case 2

**FIGURE 4:**
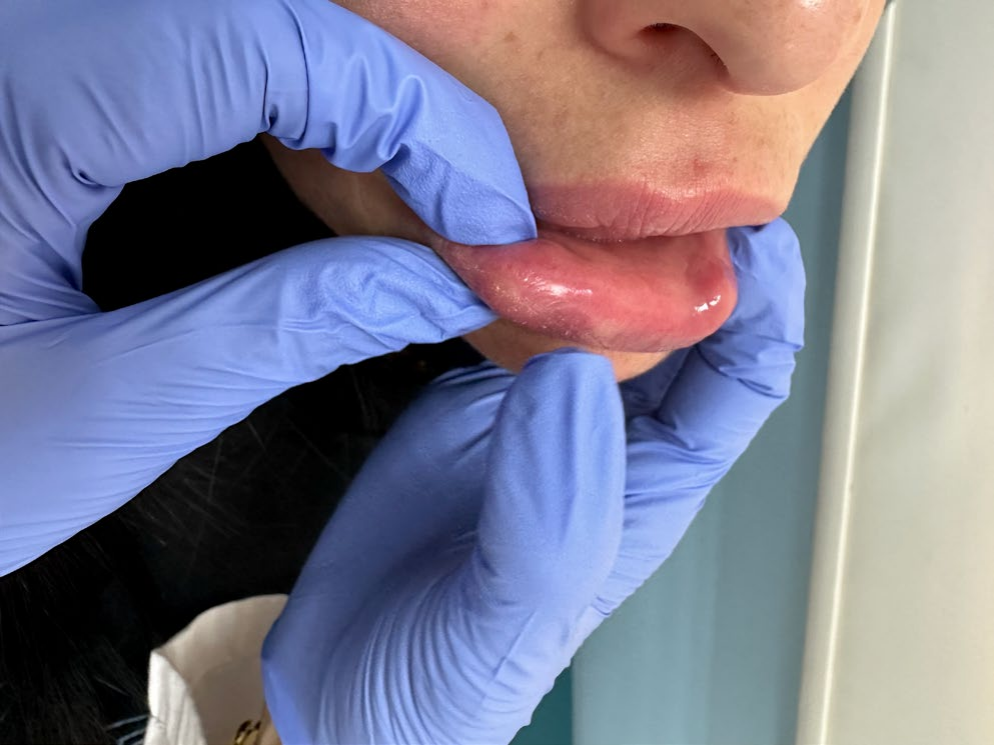
Clinical photograph of case 2 demonstrating ill‐defined areas of edema within the lips.

A 38‐year‐old Lebanese female patient presented with edema in her jaw, nasolabial folds, medial and lateral cheeks, temples, and lips (Figure 4), which had occurred repetitively over the past 1.5 years. Two years prior, she had received HA filler injections in these areas by another practitioner, but the exact filler type was unknown. Her symptoms began 6 months later, characterized by recurrent episodes of swelling and reduced range of motion, particularly in the lower half of her face. It is worth mentioning that the patient also reported having been injected with permanent fillers in her lips several years earlier, and having received two doses of a COVID‐19 mRNA vaccine 1 month after her HA filler injections and 5 months prior to the start of her symptoms.

Although she received numerous treatments at another facility, including several courses of oral antibiotics and hyaluronidase injections, she continued to experience recurrent episodes of swelling. She was also prescribed multiple courses of oral steroids, which provided temporary relief but were followed by the return of symptoms within weeks. A magnetic resonance imaging (MRI) was subsequently conducted and revealed filler material infiltrating the masseter muscles, subcutaneous tissue in bilateral temples, buccal fat pad, and the upper and lower lips, despite all the aforementioned treatments.

**FIGURE 5:**
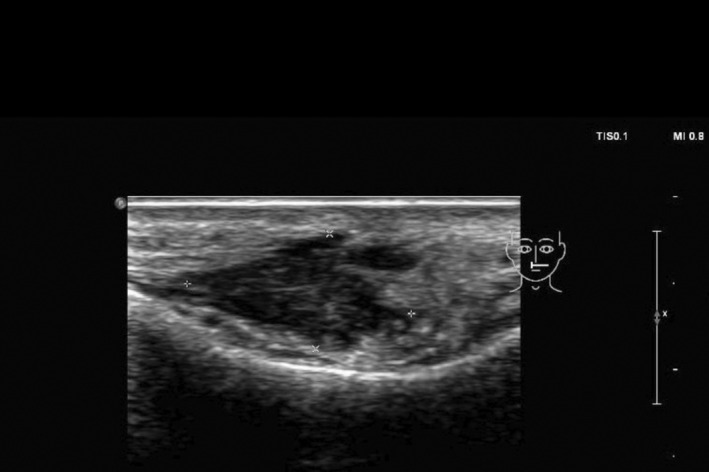
Ultrasonographic findings in patient 2 demonstrate ill‐defined, hypoechoic filler deposits within the deep subcutaneous fat in multiple locations, sometimes reaching the skeletal muscles.

Given her extensive history and failure to respond to multiple treatments, the author requested an ultrasound (Figure 5) and opted to assist the radiologist during the procedure in order to accurately visualize the sites of the filler deposits, which in turn will aid in the treatment process. Consequently, the filler was dissolved by precisely injecting hyaluronidase into the affected anatomical areas at a concentration of 150 IU/mL. The patient experienced improvement within 48 h, achieving complete resolution of her symptoms by her follow‐up appointment 1 week later. Since then, she has reported no recurrence of swelling.

### 2.3 Case 3

**FIGURE 6:**
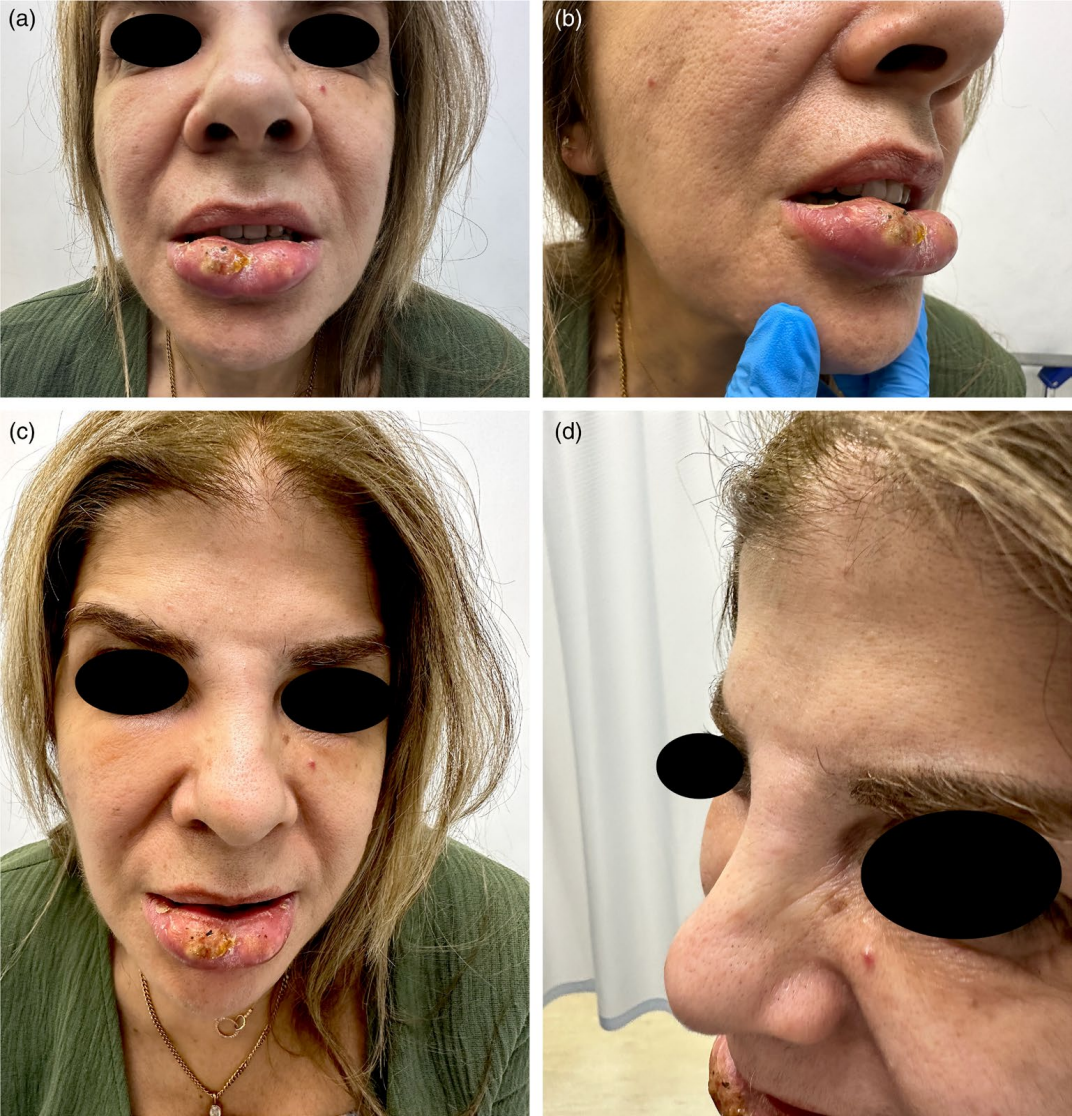
Clinical photographs of case 3 showing severe edema of the lips, crusting, purulent discharge, and abscess formation (a, b); as well as edema of the glabellar region (c, d).

**FIGURE 7:**
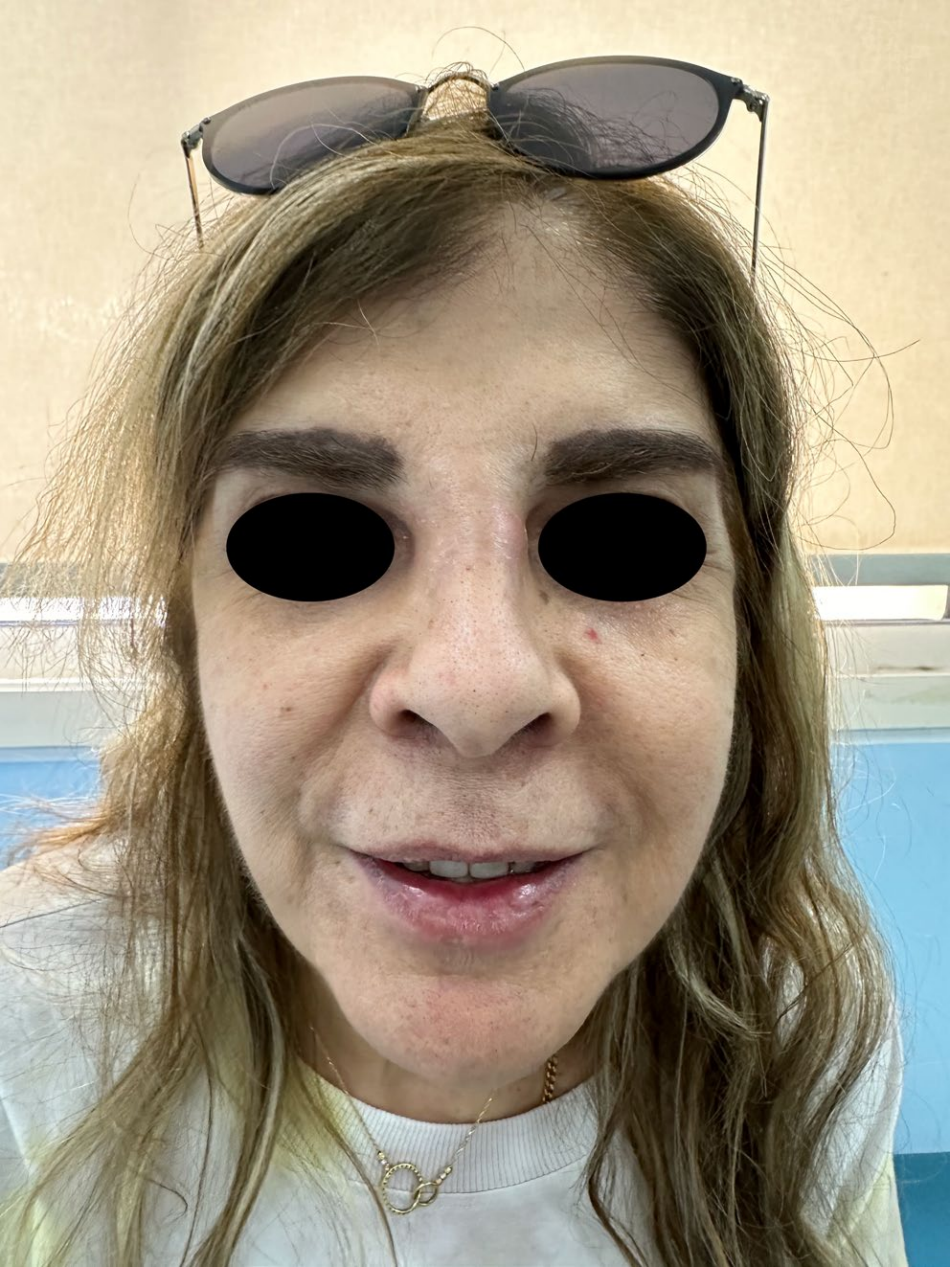
Clinical improvement seen in patient 3 at 2 weeks follow‐up.

A 60‐year‐old Lebanese female patient presented to the emergency department (ED) with severe swelling and pain in her lips, honey‐colored crusting, and multiple collections and abscesses that were oozing a green, purulent discharge (Figure 6a,b). She also had some mild swelling over the glabellar region (Figure 6c,d). These symptoms had begun a few days prior to presentation and progressively worsened over time. Three weeks earlier, she had received HA soft tissue filler injections in her lips from an unlicensed practitioner as well as botulinum toxin injections in the forehead, followed by HA injections in the glabellar region a week later with a botulinum toxin touch‐up. The type of HA filler used was Art Filler Volume (FILLMED). Two weeks after the filler injections, her lips began to swell significantly, resulting in severe pain and generalized fatigue, although no fever was recorded. She was prescribed 1 g of amoxicillin‐clavulanic acid to be taken twice daily 3 days before her ED visit, with only slight improvement. Upon her arrival, incision and drainage of the lip collections were performed, and a culture was obtained from the purulent discharge. Oral ciprofloxacin was incorporated into her treatment plan, and she was instructed to continue both antibiotic courses and return for a follow‐up a week later. At her follow‐up visit, her symptoms had greatly improved; however, she still exhibited some persistent edema in both her lips and glabella. To address this, a total of 300 U of hyaluronidase was injected into both areas, and she was prescribed a short course of oral steroids. A follow‐up appointment 5 days later revealed complete resolution of all her symptoms (Figure 7). To note, the culture results taken while the patient was on amoxicillin‐clavulanic acid showed no growth.

## 3 Discussion

Delayed‐onset adverse reactions to HA soft tissue fillers are becoming increasingly recognized as a significant concern, particularly with the rise in the use of these fillers as minimally invasive cosmetic procedures in recent years. Such reactions can manifest as cyclic or constant erythema and edema, as well as late‐onset inflammation or nodules [9]. The incidence of late‐onset nodules in a recent retrospective study has been reported as 1% per patient [10]. Nodules are generally categorized as either inflammatory or non‐inflammatory in origin. Non‐inflammatory nodules often occur immediately after treatment and are generally attributed to improper filler placement; they may not be extensively documented in the literature since they usually respond well to observation, massage, or hyaluronidase treatment [11]. In contrast, late‐onset inflammatory nodules can result from a number of different factors, including foreign‐body reactions, immune‐mediated delayed hypersensitivity reactions, and infections [12]. These etiologies are often overlapping and may co‐exist in the same patient. Clinical opinions differ on the post‐injection time frame that defines “delayed‐onset”; however, a commonly agreed‐upon consensus is a reaction that occurs more than 2 weeks after injection [13, 14].

Foreign‐body granulomas following filler injections are infrequent, with previous studies reporting an incidence between 0.02% and 2.8% [15, 16]. The underlying pathophysiology is an immune response directed against a foreign material that evades phagocytosis and degradation by macrophages. The body attempts to encapsulate and isolate this exogenous substance, which resists enzymatic breakdown. The engulfed material is thus sequestered by aggregates of activated macrophages assuming an epithelioid morphology, leading to the release of pro‐inflammatory cytokines that further recruit additional macrophages, which eventually fuse to form multi‐nucleated giant cells, a hallmark of granulomas [1, 4, 12]. Confirmation of foreign‐body granulomas can only be achieved histologically to distinguish such reactions from non‐granulomatous nodules [1, 9, 12], as demonstrated in our first case. Ultimately, activated macrophages may draw in fibroblasts and signal them to produce collagen, which helps to explain the fibrosis that is observed in long‐standing granulomas. This immune response is a systemic process and, as such, multiple sites may be affected simultaneously. Several factors can increase the risk of developing such reactions, including larger injection volumes, the use of non‐biodegradable fillers, the degree of cross‐linking, and prior infections or trauma at the injection site [1, 12]. A foreign body granulomatous reaction should be suspected when nodules appear several months after injection or when they do not respond to standard treatment approaches. The mainstay treatment method for foreign‐body granulomas is intra‐lesional corticosteroid [4], as exemplified in our first patient. Other less effective treatment options include oral corticosteroids and hyaluronidase injections. Surgical excision may be considered as a last resort if the granulomas are few in number. For long‐standing fibrotic lesions that do not respond to steroids alone, more aggressive treatments, such as intra‐lesional 5‐fluorouracil, can be utilized, as it has shown effectiveness due to its anti‐inflammatory and anti‐fibrotic properties [12].

As noted previously, not all nodules are granulomatous in nature. Other types of inflammatory nodules that occur after filler injections fall under the umbrella term “delayed inflammatory reactions” (DIRs). DIRs can manifest in various forms, such as inflammatory nodules or persistent intermittent delayed swelling (PIDS) [17]. The exact etiology of DIRs is multifactorial and includes biofilm formation and/or a delayed type IV hypersensitivity (DTH) reaction. These mechanisms may be intertwined, making it difficult to distinguish between them [18]. Multiple pathophysiological factors have been identified as contributors to DIRs and they involve the interaction between the patient's immune profile at the time of the reaction, the physiochemical properties of the filler, and the degree of bacterial contamination at the time of injection [12]. Potential triggers highlighted in the literature include recent viral infections, dental procedures, cutaneous surgeries or laser treatments, local trauma, autoimmune disorders, drug interactions, COVID‐19 infections or vaccinations, bacterial inoculation, and HA breakdown byproducts [2, 5, 7, 18]. Although native HA is generally not immunogenic, the cross‐linking that enhances the stability and durability of the molecule predisposes the filler to become more immunogenic [2, 7]. In addition to cross‐linking, HA fragment size may also influence inflammation, as low molecular weight HA (LMW‐HA) has been associated with pro‐inflammatory effects, in contrast to high molecular weight HA (HMW‐HA) [2, 12]. Additionally, Vycross cross‐linking technology used by Allergan may contribute to a filler's immunologic properties through the release of pro‐inflammatory LMW‐HA during the breakdown of HA gels [12, 18]. The combination of different filler products has also been shown to play a role in DIRs [18].

The recent application of ultrasound imaging in evaluating and managing filler complications has proven to be highly effective [19], as it allows practitioners to assess the volume, location, and depth of the injected filler, thus enabling them to tailor treatment strategies. It can also guide the administration of hyaluronidase [7, 9, 18], as demonstrated in our second case. Furthermore, ultrasounds provide valuable information about the nature of filler reactions (e.g., the presence of an abscess) and allow for real‐time monitoring of treatment responses [12]. Our second patient exemplifies the benefits of using ultrasounds in managing delayed filler reactions: the same treatment (hyaluronidase injections) was initially ineffective when performed blindly but was proven to be effective when the injections were targeted to the sites of filler deposits identified by ultrasonography. This patient initially presented with persistent yet intermittent swelling, a phenomenon referred to in the literature as PIDS [17]. In this context, “persistent” indicates that the condition spans several months or extends over a prolonged period of time, whereas “intermittent” describes its episodic nature with recurrent flare‐ups interspersed with periods of remission. This condition is characterized by non‐pitting edema along the sites of HA injections, which may be erythematous or not, localized or diffuse, ill‐ or well‐defined, and typically manifests as recurrent swelling episodes over an extended duration. Palpable and well‐defined solid nodules are usually not seen. Ultrasonically, there are detectable areas of HA filler deposits—but no solid nodules–that correspond to the edematous regions clinically [17], as was seen in our patient. These findings are often accompanied by a diffuse thickening and increased echogenicity of the subcutaneous tissue [17]. In her case, several factors may have contributed to the development of a DIR to HA fillers, including the use of different filler products (both HA and an unknown permanent filler), the potential formation of a biofilm, and possibly her mRNA COVID‐19 vaccination, which has been linked to similar reactions in previous studies [20, 21]. One key therapeutic option for managing DIR is the injection of hyaluronidase to dissolve and completely get rid of the filler [1, 5, 7, 8, 12, 18], especially when delayed‐type hypersensitivity reaction is suspected, and after ruling out the possibility of an active infection. This approach proved effective in her case, particularly when administered correctly.

Any procedure that disrupts the skin's surface carries with it a risk of infection, and injecting soft tissue fillers is no exception. Infections are reported as the second most common complication following filler injections [7]. The risk of infection does not seem to depend on the type of filler used, but rather on breaks in sterile technique [7]. Acute infections, usually presenting as acute inflammation, tender lumps, or abscesses at the injection site, are often caused by common organisms present on the skin such as *Streptococcus pyogenes* or *Staphylococcus aureus* [7, 22]. Mild cases are usually effectively treated with oral antibiotics such as amoxicillin‐clavulanic acid, azithromycin, clarithromycin, ciprofloxacin, or tetracyclines [12, 22]. Other less frequent infectious etiologies include viral, polymicrobial, or fungal organisms, including *Candida* species [7]. If left untreated, all of these organisms may lead to sepsis, particularly in older individuals or in patients with compromised immune systems [22]. Periorbital and midfacial infections require close monitoring for potential intracerebral complications. Delayed infections—occurring 2 or more weeks after filler injections—may be caused by atypical mycobacteria [7, 18, 22]; these are usually difficult to diagnose and often hard to treat. For this reason, it is advisable to obtain a bacterial culture and antibiogram to guide appropriate antibiotic therapy. Non‐tuberculous mycobacterial infections have been reported 2–6 weeks post‐injection and were often linked to the use of ice water before and after the procedure [18]. Accordingly, it is recommended to avoid applying creams or washing the face for at least 8 h after filler injections [18]. In the presence of an abscess or a fluctuating mass, incision and drainage should be performed, and the material expressed should be sent for culture and sensitivity testing [1, 2, 5, 7, 22], as applied in our third case. Hyaluronidase should be avoided until the infection is cleared, as it may facilitate the spread of pathogenic organisms to surrounding tissues [2, 7, 18, 22].

It is important to understand that a positive culture result and visible signs of infection do not rule out the possibility of a delayed‐type hypersensitivity reaction; this is because such reaction may coexist with other types of DIRs, including infections and foreign body granulomas [8]. Therefore, it is essential to first address any underlying infectious etiologies when suspected, and then consider additional treatments such as oral steroids or hyaluronidase injections if some symptoms persist, as demonstrated in our third case. In this particular patient, although signs of infection were evident, the culture results were negative. This could be accounted for by two possible theories: either the pathogenic organism failed to grow due to the patient's concomitant use of amoxicillin‐clavulanic acid, or the patient may have had a delayed infection caused by atypical mycobacteria, which did not grow on standard culture media. The latter scenario would explain the significant improvement observed after adding ciprofloxacin to the treatment regimen. This case also underscores the importance of selecting a licensed injector for cosmetic procedures, as illegal practitioners may not adhere to proper aseptic techniques, thereby increasing the risk of post‐filler infections.

To prevent infections during filler injections, strict adherence to aseptic and clean practices should be emphasized. Complete removal of a patient's makeup and thorough cleansing of the entire face are essential [7, 23]. Every needle pass through the skin carries a risk of bacterial contamination. To minimize this risk, antiseptic solutions such as chlorhexidine gluconate (CHG) and isopropanol are recommended. CHG, in particular, is the preferred disinfectant as it has been linked to the decreased incidence of infection in minimally invasive cosmetic procedures such as fillers [7, 23]. Betadine may be used as an alternative disinfectant when injecting near the eyes. It is advisable to apply the antiseptic across the whole face and to initiate treatment in areas away from the nose and mouth, as these regions are more prone to bacterial contamination [7]. It is also crucial to assess a patient's prior injection history and infectious history, as this may provide insights on a patient's particular risk [23]. Finally, patients should be instructed not to apply cosmetic products or tap water on the face for at least 8 h post‐filler injection [18].

## 4 Conclusion

Aesthetic treatments using soft‐tissue HA fillers remain one of the most sought‐after cosmetic procedures. When administered by skilled and experienced practitioners, filler injections are generally regarded as safe; however, complications may still occur. Identifying these complications is crucial, as is prompt intervention. This article offers valuable insights and practical recommendations to help clinicians effectively manage delayed HA filler reactions. Imaging is playing an increasingly important role in diagnosing non‐ischemic filler complications and is expected to drive future innovations aimed at improving patient outcomes. To reduce the risk of complications, filler injections should only be performed by licensed, trained professionals who adhere to aseptic protocols, have a strong understanding of facial anatomy, carefully select appropriate patients, and use the most suitable products and injection techniques.

## Author Contributions

Zeina Tannous and Yara Saad were involved in collecting the data and information needed and in the direct care of the patients. Yara Saad drafted the manuscript, and both authors were involved in critically revising the manuscript for fundamental intellectual contribution. Zeina Tannous and Yara Saad reviewed and approved the final version of the manuscript.

## Ethics Statement

Study protocol was reviewed and the need for approval was waived by the Lebanese American University Institutional Review Board (IRB). Written informed consent was obtained from the patients for publication of the details of their medical cases and any accompanying images.

## Conflicts of Interest

The authors declare no conflicts of interest.

## Data Availability

All data underlying the results are available as part of the article and no additional source data are required. Further enquiries can be directed to the corresponding author.
